# Killing of Gold Nanorods-Loaded Human Cardiac Fibroblasts Mediated by Photo-Thermal Activation

**DOI:** 10.1021/acsomega.5c12235

**Published:** 2026-02-04

**Authors:** Erica Floris, Flaminia Pompeo, Vittorio Picchio, Selenia Miglietta, Vincenzo De Mei, Claudia Cozzolino, Francesca Icolaro, Vincenzo Petrozza, Giacomo Frati, Francesca Petronella, Isotta Chimenti, Francesca Pagano, Luciano De Sio

**Affiliations:** † 9311 Department of Medical Surgical Sciences and Biotechnologies, Sapienza University of Rome, Corso della Repubblica 79, 04100 Latina, Italy; ‡ Institute of Crystallography, National Research Council of Italy, CNR-IC, 00010 Montelibretti (RM), Italy; § Department of Angio-Cardio-Neurology, IRCCS Neuromed, Via Atinense 18, Pozzilli, 86077 Isernia, Italy; ∥ Department of Anatomy, Histology, Forensic Medicine and Orthopaedics, Sapienza University of Rome, Via A. Borelli 50, 00161 Rome, Italy; ⊥ Maria Cecilia Hospital, GVM Care & Research, Via Corriera 1, 48033 Cotignola (RA), Italy; # Institute of Biochemistry and Cell Biology, National Research Council of Italy, Via E. Ramarini 32, 00015 Monterotondo Scalo (RM), Italy

## Abstract

Cardiac fibrosis is a pathological process associated with several heart diseases, characterized by extracellular matrix deposition by cardiac fibroblasts. Photothermal therapy is a minimally invasive medical treatment that uses nanomaterials to convert external light into localized heating. On these premises, we report an innovative approach that exploits gold nanorods (AuNRs)-mediated plasmonic photothermal therapy (PPTT) for selective ablation of AuNR-loaded human cardiac fibroblasts (hCFs). Cellular uptake is confirmed by treating hCFs with AuNRs, irradiating them with an 808 nm continuous-wave (CW) laser, and performing ultrastructural electron microscopy analysis. Measurements of extinction spectra, temperature variation, and cell viability were conducted. Results show that hCFs internalize AuNRs effectively without cytotoxicity, and extinction spectra reveal a concentration-dependent redshift of the longitudinal plasmon bands. Upon laser irradiation, AuNR-loaded hCFs experience a temperature increase, leading to cell death in proportion to AuNRs concentration. In coculture systems, AuNR-loaded hCFs are ablated while the viability of surrounding nonloaded cells (fibroblasts or cardiomyocytes) remains unaffected, demonstrating that hCFs can internalize AuNRs and be effectively ablated through PPTT. Our results highlight the potential of AuNRs for targeted cardiac cell ablation with minimal impact on adjacent cells.

## Graphical abstract

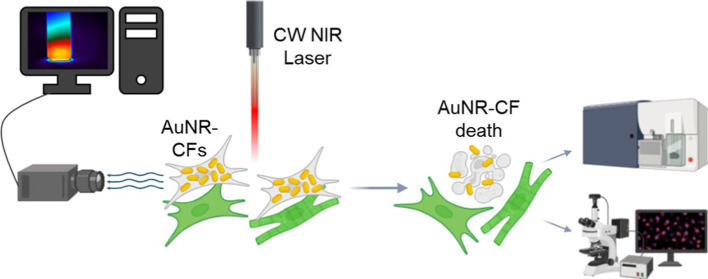

## 1 Introduction

Photothermal therapy is a minimally invasive cancer treatment that uses photosensitizer molecules that efficiently convert light energy into heat. When exposed to a specific light source, typically in the near-infrared (NIR) range, these photosensitizers absorb light and convert it into heat, raising the temperature in targeted tumor areas. This localized heating can effectively destroy cancer cells while minimizing damage to surrounding healthy tissue.^1^ Among the different photosensitizers, gold nanoparticles (AuNPs) have peculiar and tunable physical-chemical features, for which they have recently found application in various biomedical fields, from imaging and diagnostic techniques to delivery systems for specific conjugated molecules and therapeutic interventions.^2,3^ . Plasmonic photothermal therapy (PPTT) is a promising application in this latter case. This noninvasive, drug-free therapy exploits the photothermal heating of irradiated AuNPs to ablate detrimental/pathological cells that actively internalize the particles. Nanomaterials such as AuNPs are characterized by a high surface/volume ratio and, in addition, are efficient thermo-optical transducers. For this reason, one of the most promising biomedical applications of plasmonic NPs is PPTT. When a plasmonic NP is illuminated at the resonant frequency, strong absorption of light occurs, which is converted into heat. Specifically, light energy is converted into heat through a sequence of well-defined steps: electron excitation, rapid thermalization, and subsequent energy transfer to the lattice. These steps are precisely described by the two-temperature model, which explains the process on ultrafast time scales (femtoseconds to picoseconds).^4^ Differently, the same phenomena can be interpreted more simply through the Joule effect, which offers a simple yet accurate explanation of the same mechanisms on time scales of the order of seconds.^5^ Thus, plasmonic NPs can be nano sources of localized heat, which allows irreversible cell damage and death of the cells which internalize the particles, thus allowing the ablation of targeted cells in tissues in a precise and controllable manner.^6^ AuNP-mediated PPTT has been applied mainly as a minimally invasive treatment against cancer,^7^ alone or in combination with established cancer therapies.^8^ In particular, cancer cells are characterized by the enhanced permeability and retention (EPR) effect, which facilitates NP preferential accumulation in solid tumors rather than in the surrounding healthy tissues.^9^ In addition, tumor tissues often have tortuous and abnormal vasculature, which makes it difficult to dissipate heat, thereby increasing their sensitivity to hyperthermia compared with healthy tissues.^10^ Hence, for the peculiar ability of cancer cells to uptake AuNPs, combined with the physical features of solid tumors, cancer therapy has been the main suggested application exploiting AuNPs’ photothermal features in biomedicine. Several studies show AuNP-based PPTT has been successfully tested for breast cancer,^11^ prostate cancer,^12^ lung cancer,^13^ and brain cancer reduction therapies.^14,15^

During the last years, the concept of AuNP-mediated PPTT has been extended to the design of treatment approaches for diseases other than cancer, where ablation of specific cells is desirable and offers an effective treatment option. AuNP-mediated PPTT has been used against bacteria, such as *Pseudomonas aeruginosa* ^16^ and *Staphylococcus aureus*,^17^ demonstrating its effectiveness and applicability in the treatment of drug-resistant infections. One specific study has shown successful PPTT for the treatment of liver fibrosis in mice mediated by gold nanorods (AuNRs), selectively targeting hepatic stellate cells through anti-PDGFRβ antibody-conjugated AuNRs.^18^ Fibrosis represents a main pathogenetic mechanism in multiple organs and diseases, including cardiovascular diseases (CVDs), which are currently the leading cause of death in the world, and have a significant impact on society and economics.^19^ Cardiac fibrosis is a condition commonly found in a wide range of CVDs.^20,21^ In some cases, fibrosis is the result of a localized reparative process in response to injuries that have led to cardiomyocyte death, resulting in tissue replacement with a nonfunctional scar, such as in the case of a myocardial infarction.^22^ In other cases, the fibrotic process is interstitial, consisting of continuous cardiac fibroblast (CF) stimulation due to chronic conditions and inflammation, such as in the case of pressure or volume overload,^23^ diabetes, metabolic syndrome and obesity.^24^ Moreover, myocardial fibrosis can be associated with genetic cardiomyopathies.^25^

Independent of its cause, the fibrotic process is a maladaptive, yet necessary response to maintain the structural integrity of myocardial tissue. Nonetheless, cardiac fibrosis progressively impairs the heart’s contractile capacity and alters the cellular microenvironment, eventually leading to cardiac muscle dysfunction and heart failure.^26^ The central effectors in the fibrotic process are CFs, which are the main producers of extracellular matrix (ECM) proteins in both homeostasis and disease.^27^ CFs are a heterogeneous cell population characterized by distinct functional states, each with specific surface markers and either pro-inflammatory and pro-fibrotic or trophic and beneficial to other cell types in the tissue.^28,29^ In fact, under physiological conditions, homeostatic CFs support the survival and function of different cell types by exerting protective paracrine effects on cardiomyocytes, endothelial cells, resident immune cells, and other stromal cell populations. Moreover, they regulate ECM synthesis and turnover.^30^ When stimulated, CFs undergo activation and specification into myofibroblasts, which produce large amounts of ECM proteins (particularly collagen I).^31^ Interestingly, studies in models of cardiac fibrosis have shown that a partial depletion (through genetic or immunological strategies) of activated CFs, or even of the whole CF population during the pathogenetic stimuli, leads to beneficial effects, partially reducing the extent of collagen deposition and its negative effects on cardiac function.^32,33^ Thus, the fibrotic process can happen up to a balance where it avoids organ rupture, while reducing to a minimum the overall detrimental effects on cardiac remodeling.

Despite notable progress, current therapeutic approaches for cardiac fibrosis remain limited in their ability to reverse established pathological remodeling.^34,35^ Conventional drugs, including ACE inhibitors, angiotensin receptor blockers, β-blockers, and aldosterone antagonists, provide only partial protection and act on signaling pathways that are not exclusive to fibroblasts.^36^ Targeted inhibition of pathways such as TGF-β or Smad3 may attenuate fibrosis but risks disrupting physiological repair mechanisms. Emerging regenerative and biomaterial-based strategies, including hydrogels, engineered scaffolds, and mRNA delivery systems, show potential yet remain largely experimental, with uncertain long-term outcomes. Building on this foundation, recent preclinical research has expanded the landscape of antifibrotic therapy through the repurposing of noncardiac drugs and the development of novel targeted and immunotherapeutic strategies.^35,37^ Agents such as pirfenidone, nintedanib, rapamycin, and SGLT2 inhibitors have demonstrated promising antifibrotic mechanisms, though evidence remains largely preclinical. Innovative approaches have illustrated the growing potential of precision-guided interventions. Recent studies have highlighted that modulating the distinct phenotypic features of activated CFs and myofibroblasts may help limit the progression of fibrosis. Such an approach could preserve the fibrotic response required for tissue integrity while minimizing scar expansion, thereby supporting the survival and function of parenchymal cells. Evidence from genetic depletion studies further indicates that a controlled reduction in CF numbers can have therapeutic benefits by decreasing scar formation and maintaining cardiac performance.^33^ Two recent articles showed that CAR-T cells targeting FAP-expressing activated CFs could reduce cardiac fibrosis and restore myocardial function after both pharmacological and surgical cardiac injury.^32,38^

In this study, we leverage the properties of AuNRs as efficient light-to-heat converters within the intracellular space to explore for the first time the feasibility of a PPTT approach for the ablation of CFs. For this work, we selected anisotropic AuNPs (AuNRs) for their sensitivity to changes in the refractive index of the medium and their high efficiency at converting light into heat. The morphology of AuNRs maximizes light-to-heat transduction efficiency, resulting in more effective treatment. We investigated here the active uptake of AuNRs by human primary CFs (hCFs) and the efficacy of photothermal conversion of NIR light to induce necrosis in these cells, either alone or in coculture with untreated hCFs, and in proximity to cardiomyocytes. This proof-of-concept study aims to define an AuNR-based photothermal depletion strategy targeting AuNP-loaded CFs while avoiding damage to surrounding cells.

## 2 Experimental Section

### 2.1 Optical Characterization of AuNRs

The NPs used for all experiments are AuNR800 purchased from nanoComposix (GRCN800). These AuNRs were synthesized to have the longitudinal extinction peak at λ = 808 nm. They feature a typical aspect ratio (AR) of 3.66, with a length of 55 nm ± 18 nm, a width of 15 nm ± 5 nm, and a ζ potential between −20 and −80 mV.

### 2.2 UV–vis Spectroscopy

Extinction spectra were measured with the UV–vis PerkinElmer LAMBDA 365 spectrophotometer. The extinction spectrum of the medium in which the cells were suspended was acquired as a reference sample (blank). All measurements were performed in a quartz cuvette.

### 2.3 Human Cardiac Fibroblast Isolation and Treatment

hCFs were isolated through primary explant culture from surgical biopsies of left atrial appendages during clinically indicated procedures of elective cardiac surgery, after informed consent, and according to the principles of the Declaration of Helsinki, under protocol 2154/15 approved by the Ethical Committee of “Umberto I″ Hospital, “La Sapienza” University of Rome. Explant outgrowth cells were collected 3 weeks after tissue culture establishment with mild digestion performing sequential washes with Ca^2+^-Mg^2+^ free phosphate-buffered saline (PBS), 0.48 mM Versene (Thermo Fisher Scientific, Waltham, MA, USA) for 3 min, and 0.05% trypsin–EDTA (Lonza, Basel, Switzerland) for 5 min at room temperature under visual control. Harvests were made weekly up to three times. Cells were cultured in complete explant media (CEM) [Iscove’s modified Dulbecco’s medium (IMDM) (Sigma-Aldrich) supplemented with 20% FBS (Sigma-Aldrich), 1% penicillin–streptomycin (Sigma-Aldrich), 1% l-glutamine (Lonza), and 0.1 mM 2-mercaptoethanol (Thermo Fisher Scientific)] on fibronectin (BD-Biosciences) -coated surfaces. For all treatments, cells were plated at a density of 10^4^ cells/cm^2^ and treated with AuNRs at final concentrations of 50 μM, 100 μM, and 200 μM in 20% FBS CEM for 72 h. The cells were double-washed with Ca^2+^-Mg^2+^ free PBS before every analysis performed in order to remove noninternalized AuNRs.

### 2.4 Ultrastructural Transmission Electron Microscopy

Cellular uptake of AuNRs and ultrastructural changes were verified by Transmission Electron Microscopy (TEM). After 72 h incubation with 0 μM, 50 μM, 100 μM and 200 μMAuNRs, cells were detached and transferred in the Eppendorf tubes for TEM processing. After centrifugation, at 250*g* for 5 min, the cell pellet was fixed with 2.5% glutaraldehyde (SIC, Rome, Italy) in 0.1 M PBS for 2 days at 4 °C and then rinsed with PBS. Afterward, samples were postfixed using 2% osmium tetroxide (Agar Scientific, Stansted, UK) for 2 h and rinsed again in PBS. The samples were dehydrated by exchange with ethanol, immersed in propylene oxide (BDH Italia, Milan, Italy) for solvent substitution, and embedded in epoxy resin Embed-812 (SIC). Ultrathin (80–90 nm) sections were obtained using an ultramicrotome (Leica EM UC6; Leica); for the TEM observation, the ultrathin sections were collected on 100-mesh copper grids (Assing, Rome, Italy) stained with a mix of lanthanides solution (UranyLess; Electron Microscopy Sciences, PA, USA) and lead citrate. Imaging was performed using a TEM (Carl Zeiss EM10; Carl Zeiss, NY, USA) at 60 kV, with a DEBEN XR80 AMT CCD camera (Deben, Melton, UK).

### 2.5 MTS Cell Viability Assay

MTS cell viability assay was performed to evaluate cell proliferation using Cell Titer 96 Aqueous Non-Radioactive Cell Proliferation Assay (MTS) (Promega, Madison, WI, USA). Cells were treated in 96-well plates at a density of 5 × 10^4^ cells/cm^2^ in 100 μL of culture medium with 0 μM, 50 μM, and 100 μM AuNRs. After 72 h, hCF viability was measured by adding 20 μL of combined MTS/PMS Solution to each well and incubating for 1 h. The absorbance at 490 nm was recorded using Varioskan LUX Multimode Reader (Thermo Fisher Scientific) and normalized to T0 absorbance.

### 2.6 HL-1 Cell Culture

HL-1 cardiomyocytes were maintained in Claycomb medium supplemented with 10% FBS (Sigma-Aldrich), 1% penicillin–streptomycin (Sigma-Aldrich), 1% l-glutamine (Lonza), and 0.1 mM Norepinephrine (Sigma-Aldrich), on gelatin/fibronectin-coated surfaces. For mixed-culture experiments, a GFP-expressing HL-1 (HL-1^GFP^) cell line was established and mixed with AuNR-CFs in cell suspension.

### 2.7 Generation of GFP + hCFs and HL-1

The lines expressing GFP have been generated by lentiviral transduction of hCFs. The viral particles were generated using the III generation pCCL PGK-GFP lentiviral plasmid which allows the expression of a GFP reporter from its promoter. The lentiviral particles were generated by transfecting the HEK-293T cells with the pCCL PGK-GFP, REV, MDL and VSVG plasmids using TurboFect transfection reagent (Thermo Scientific, MA, USA) in DMEM (Sigma-Aldrich) supplemented with 10% FBS (Sigma-Aldrich). The transfection was performed according to the manufacturer’s standard protocol. The media containing the lentiviral particles was collected after 24 h, and the infection of hCFs and HL-1 was performed using a spin-infection protocol. Briefly, 1 × 10^5^ cells were seeded in 6-well plates and spun at 1800 rpm at 34 °C for 45 min with 1.5 mL of lentiviral preparation supernatant. The cells were kept in a humidified chamber incubator for a further 24 h in the presence of the lentiviral particle containing media. The media was changed, and fluorescence intensity was checked after 48 h. The infection efficiency was 95–100%.

### 2.8 AuNR-hCFs/hCFsGFP Mixed Coculture

Cells were seeded on an 8-well culture slide (Falcon) at a final cell density of 7 × 10^3^ cells/cm^2^. This cell culture was composed of 50% hCFs pretreated with 100 μM AuNRs for 72 h (AuNR-hCFs), and 50% nontreated GFP-expressing hCFs (hCFsGFP). Four hours after seeding the mixed culture, the chambers were removed, and PBS 2% FBS was added to the cells. The slide was covered with a cover glass and illuminated.

### 2.9 Thermo-Optical Setup for Cell Suspension in Quartz Cuvette

The thermo-optical setup realized to evaluate the effects of NIR light on the system made by fibroblasts and AuNRs is illustrated in Figure 4a. A quartz cuvette with dimensions of 1 × 1 × 3.5 cm^3^, featuring four optically transparent walls, was utilized for the experiment. The sample (1 mL), contained in a quartz cuvette, was illuminated from the top with a continuous-wave (CW) diode laser (Coherent Powerline) operating at 808 nm, corresponding to the longitudinal plasmon resonance band of the AuNRs solution. To prevent any movement during the illumination process, the cuvette was securely attached to the surface of a sample holder using adhesive tape. The sample holder, in turn, was firmly mounted on an optical table. Cells suspended in a medium tend to settle quickly to the bottom of the cuvette. Therefore, we chose to illuminate the cuvette from above (at the interface between the sample and the air) and measure temperature changes on one side of the cuvette (the thermal imaging camera is positioned at a 90° angle to the laser). The distance from the laser source to the surface of the solution was set to 12 cm. This setup ensures uniform irradiation throughout the experiment. The power density of the laser was set at 28.3 W/cm^2^, following preliminary measurements to optimize measurement times without causing excessively rapid temperature increases. The power density was evaluated and selected to optimize the photothermal effects of the AuNRs in this specific configuration and reach temperatures high enough to obtain the killing effects on the irradiated cells.^39^ The need to use such high power densities is likely related to the fact that after washing the cells twice in PBS, the noninternalized AuNRs are removed. This significantly decreases the concentration of AuNRs within the cells, making it necessary to increase the laser power density to achieve photothermal ablation of cardiac fibroblasts. Temperature measurements at the cuvette–air interface were performed using a FLIR A700 thermal camera (FLIR Systems, Wilsonville, OR, USA) equipped with a close-up IR objective. The camera was positioned perpendicular to one of the cuvette walls, acquiring lateral thermal images from a distance of 5 cm from the cuvette surface (Figure 4a).

The thermal imaging camera is connected to a computer equipped with the Research-IR software, which allows the variation in the average surface temperature of the sample in both the spatial and temporal domains. A square region of interest (ROI) was chosen, corresponding to the cross-section of the cuvette to measure the average temperature values reached by the sample, thus excluding from the calculation of the average areas framed by the thermal camera that was irrelevant for the measurement. The illuminating protocol was: 10 s laser off, 10 min laser on, 1 min laser off. Ten minutes was chosen as the lighting time after a series of preliminary measurements.

### 2.10 Thermo-Optical Setup for Adherent Cells on Culture Slides

A schematic of the photothermal setup used for the mixed coculture sample, contained in a microscope slide covered with a cover glass, is shown in Figure S2.

A CW fiber-coupled laser emitting at 808 nm (MDL-III-808-2W), corresponding to the longitudinal plasmon resonance band of the AuNRs, irradiated the coculture sample through the glass slide from below. The laser power density was set to 30.5 W/cm^2^, as optimized through preliminary measurements to allow sufficiently long irradiation times while maintaining controlled temperature increases. The resulting temperature rise and its spatial distribution were monitored from above using a FLIR A700 thermal camera (FLIR Systems, Wilsonville, OR, USA) equipped with a close-up infrared objective. The thermal camera was interfaced with a computer running ResearchIR software, enabling quantitative analysis of the sample’s average surface temperature as a function of both space and time.

A square region of interest (ROI), corresponding to the laser spot on the sample, was selected to measure the average temperature reached by the sample. This approach excluded irrelevant areas captured by the thermal camera from the average temperature calculations, ensuring accurate thermal profiling within the laser-illuminated area. The illumination protocol consisted of the following steps: 10 s with the laser off, 10 min with the laser on, and 1 min with the laser off. The 10 min illumination period was chosen based on preliminary tests to optimize heating duration.

### 2.11 Flow Cytometry Analysis

Flow cytometry was used to assess the cell viability of hCFs irradiated in a quartz cuvette. According to the manufacturer’s guidelines, cells were stained with Annexin V-PE and Propidium Iodide (BD Bioscience). All data acquisition was performed on a FACS-Aria II platform (BD Biosciences) equipped with FACSDiva software (BD Bioscience), which was also used to calculate compensation parameters. All flow cytometry data were analyzed with FlowJo software (FlowJo LLC).

### 2.12 Viability/Cytotoxicity Assay for Fluorescence Microscopy

hCFs were plated on an 8-well culture slide (Falcon) at a cell density of 7 × 10^3^ cells/cm^2^, using different culture conditions: 100% hCFs pretreated with 100 μM AuNRs for 72 h; 100% nontreated hCFs; 50% hCFs pretreated with 100 μM AuNRs for 72 h and 50% nontreated GFP + hCFs. Four hours after seeding, the chambers were removed, PBS 2% FBS was added on the cells, and the slide was covered with a cover glass. After irradiation for 10 min with an 808 nm CW laser beam, cells were incubated for 30 min in the dark at room temperature in 4 μM EthD-III (*E*_*x*_/*E*_*m*_: 532/625 nm) and 2 μM Calcein AM (*E*_*x*_/*E*_*m*_: 494/517 nm). After staining, the cells were washed with PBS, and the fluorescence intensity was imaged using a Nikon Eclipse Ni microscope equipped with the VICO system and NIS-Elements AR 4.30.02 software (Nikon Corporation).

### 2.13 Statistical Analysis

Statistical analysis was performed using GraphPad Prism 8 software. All results are presented as mean value ± standard error of the mean. Significance of difference between two groups was determined by two-sided Student’s *t*-test. When 3 or more groups were specifically intercompared, the parametric or nonparametric (as appropriate) one-way ANOVA test followed by the Bonferroni correction or uncorrected Dunn’s test for multiple comparisons, respectively, were used. A value of *p* < 0.05 was considered to be significant. ***: *p* < 0.001. ****: *p* < 0.00001.

## 3 Results and Discussion

### 3.1 Morphological and Optical Characterization of AuNRs

The morphology of AuNRs was first investigated by ultrastructural transmission electron microscopy (TEM) to assess their dimensional features. From the statistical analysis of TEM micrographs (inset of Figure 1), the average dimensions of AuNRs are 53 nm ± 20 nm length, 15 nm ± 5 nm width, in agreement with the data sheet from the seller. Figure 1 clearly highlights the presence of the typical spectroscopic fingerprints of AuNRs that exhibit the transverse and longitudinal plasmon bands at 520 and 798 nm, respectively.

**Figure 1:**
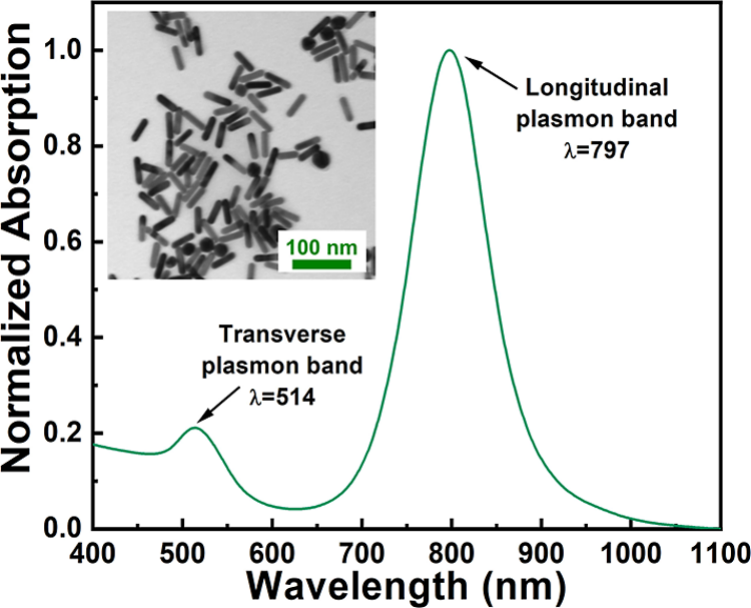
Morphological and optical features of AuNRs. Extinction spectra of the AuNRs highlighting the characteristic transverse and longitudinal plasmon bands. In the inset, a representative TEM image of the AuNRs is shown.

We further characterized the photothermal properties of the AuNRs dispersed in water at varying concentrations. Specifically, the AuNR dispersions at increasing concentrations (50 μM, 100 μM, and 200 μM) were illuminated at increasing laser power densities to demonstrate the linear correlation between the maximum temperatures reached during illumination and the laser power density. For this purpose, shorter illumination times were used than those described in the illumination protocols for AuNR-loaded cells. The time/temperature profiles obtained showed increased maximum temperature in each solution with increasing laser power densities (Figure 2a–c). The calculation of temperature increase (Δ*T*_max_) as a function of laser intensity (Figure 2d–f) demonstrated the typical linear dependence of temperature increase on laser intensity.^40^ As expected, the temperature increase is correlated with AuNR concentration for a given laser intensity. When irradiated for 170 s at 14.3 W/cm^2^, Δ*T*_max_ values of 7 °C (Figure 2a,d), 12 °C (Figure 2b,e), and 19 °C (Figure 2c,f) were recorded for AuNR concentrations of 50 μM, 100 μM, and 200 μM, respectively.

**Figure 2:**
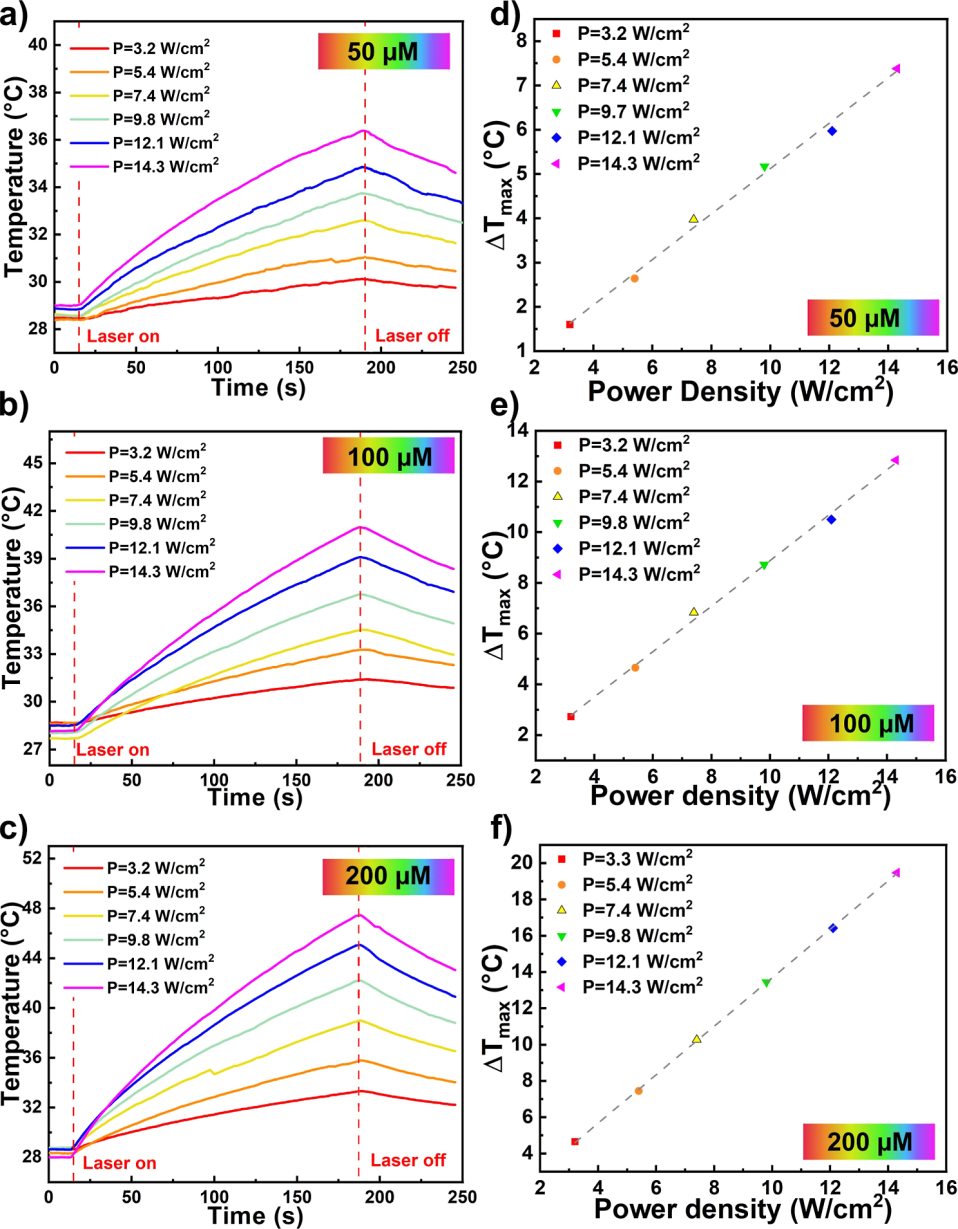
Photothermal characterization of AuNR solutions. Representative temperature–time profiles of AuNR suspensions in PBS recorded during 170 s of irradiation with an 808 nm CW diode laser at different power densities for different AuNR concentrations: 50 μM (a), 100 μM (c), and 200 μM (e). Linear fits of the maximum temperature reached by the AuNR solutions in PBS after 170 s of irradiation as a function of the power density for different AuNR concentrations: 50 μM (b), 100 μM (d), and 200 μM (f).

### 3.2 Internalization of AuNRs by Cardiac Fibroblasts without Cytotoxic Effects

Primary hCFs were isolated through explant culture from surgical biopsies of left atrial appendages with an established procedure,^41^ and were treated with 50 μM, 100 μM, and 200 μM AuNRs for 72 h (Figure 3a). The biocompatibility of AuNRs was assessed using the MTS assay, which revealed similar levels of cell viability among all the AuNR-treated samples and the control after 72 h (Figure 3b), indicating the absence of cytotoxic effects due to AuNRs. In addition, cell apoptosis rates were assessed through flow cytometry analysis of AnnexinV (AnnV) and Propidium Iodide (PI) staining. The percentage of live cells, identified as AnnV-/PI-, was similar in all samples (Figure 3c,d), revealing that AuNRs exert no cytotoxic effects on hCFs. The percentage of early apoptotic and late apoptotic cells, respectively AnnV+/PI- and AnnV+/PI+, was very low in all samples, confirming that AuNR treatment does not affect cell viability.

**Figure 3:**
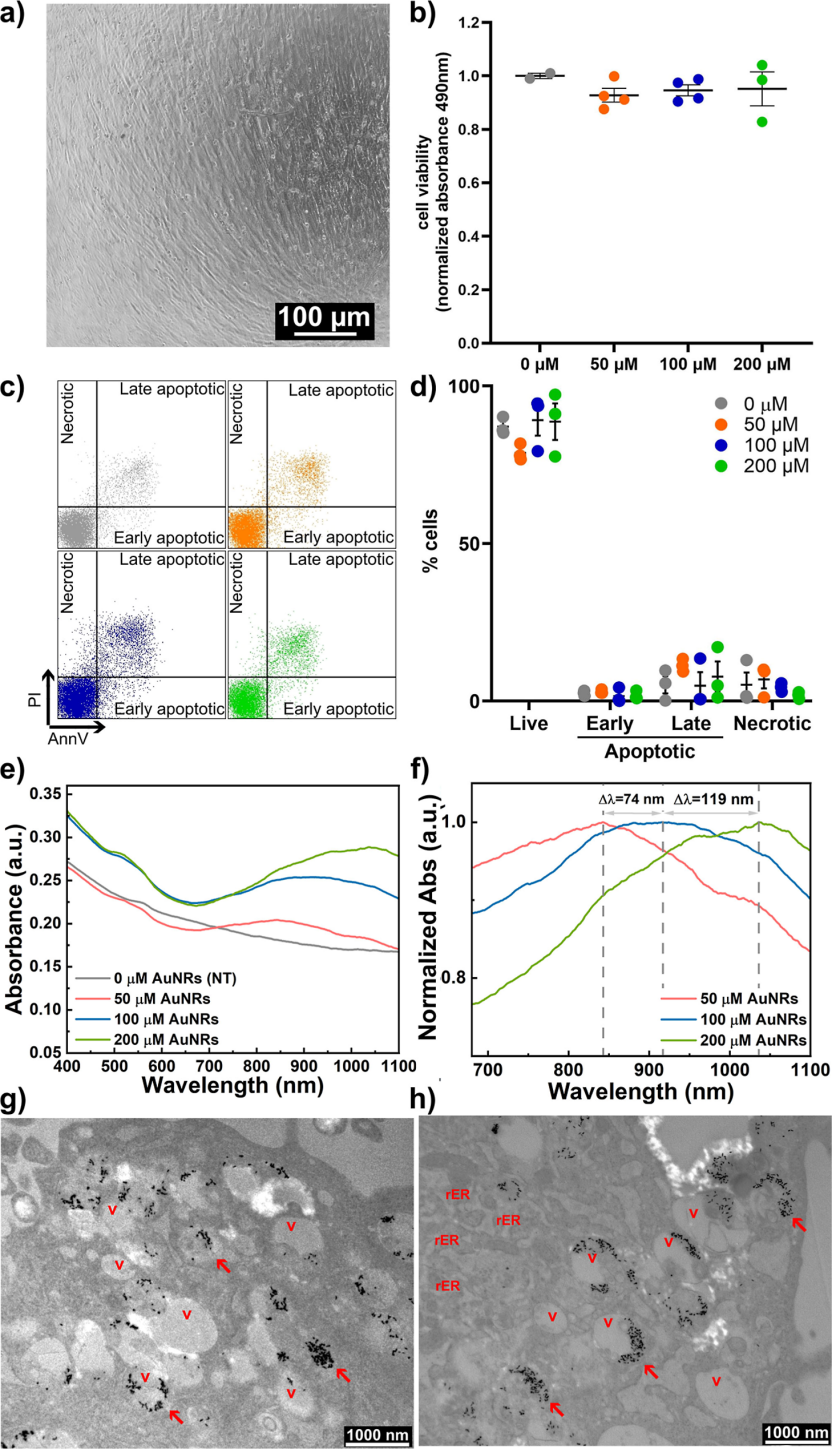
Interaction of AuNRs with cardiac fibroblasts. (a) Representative optical microscopy image of the hCF culture. (b) Dot plots of normalized absorbance at 490 nm of MTS assay for the evaluation of hCF viability after treatment with 50 μM, 100 μM, and 200 μM AuNRs for 72 h, compared with nontreated control cells (0 μM AuNRs; *n* = 3). (c) Representative dot plots of flow cytometry analysis for the evaluation of hCF viability after treatment with 0 μM, 50 μM, 100 μM, and 200 μM AuNRs for 72 h. Cells were stained with AnnexinV and Propidium Iodide for the gating of necrotic (AnnV-/PI+), late apoptotic (AnnV+/PI+), and early apoptotic cells (AnnV+/PI-). (d) Dot plots of the percentage of live, early apoptotic, late apoptotic, and necrotic cells, in the hCF culture treated with 0 μM, 50 μM, 100 μM, and 200 μM AuNRs for 72 h, evaluated by AnnV/PI staining and flow cytometry analysis. The percentage of live cells, identified as AnnV-/PI-, was similar in all samples (87.07 ± 1.58% for 0 μM, 78.77 ± 1.51 for 50 μM, 89.10 ± 4.90% for 100 μM, and 88.63 ± 5.79% for 200 μM AuNRs; *n* = 3). (e) Comparison between the extinction spectra of the four samples. It can be observed that the concentration of AuNRs in the cells varies, the longitudinal peak undergoes a red shift: λ_max_ (50 μM) = 843 nm, λ_max_(100 μM) = 917 nm and λ_max_(200 μM) = 1036 nm. (f) Normalized extinction of the samples with different AuNR concentrations. Representative ultrastructural TEM images of the AuNR-treated hCFs using 50 μM (g) and 100 μM (h) AuNRs dispersions. V: vacuole. rER: rough endoplasmic reticulum. Red Arrows: clustered AuNR sacs.

The cells exposed to increasing concentrations of AuNRs were further characterized by spectroscopic analysis (Figure 3e,f), showing a broad absorption signal associated with the longitudinal plasmon band of AuNRs at wavelengths higher than 800 nm. The intensity of this signal increased with the concentration of AuNRs. Additionally, the longitudinal peak exhibited a redshift (Figure 3f) due to changes in the local refractive index (*n*) experienced by the AuNRs in contact with hCFs (from water, *n* = 1.33, to cells, *n* = 1.4).^42^ Specifically, the wavelength corresponding to the absorption maximum for hCFs treated with 50 μM AuNRs was 843 nm, for 100 μM AuNRs it was 917 nm. For 200 μM AuNRs it was 1036 nm (Figure 3e,f). This spectroscopic behavior indicates an interaction between hCFs and AuNRs. Visual confirmation of the active cellular uptake of AuNRs by hCFs was obtained through TEM analysis, which revealed the presence of internalized AuNRs in endocytic-like vacuoles. Furthermore, TEM analysis showed no significant subcellular alterations in the presence of cellular uptake of AuNRs, and cells displayed a regular arrangement of organelles with mitochondria (m) (Figure S1a). The endoplasmic reticulum (ER) was primarily rough type (rER), with normal cisternal distension observed. Notably, numerous endocytic-like vacuoles (V) containing clustered AuNRs (red arrows) were scattered throughout the cytosol, typically appearing as sphere-shaped sacs with electron-dense contents (Figure 3g,h). Cellular morphology appeared to be particularly preserved despite the treatment, with no signs of apoptosis or autophagy identified. Further confirmation of the internalization of AuNRs after 72 h of incubation was provided by the linear dependence between the maximum temperature reached by the samples and the AuNR concentration (Figure S1b–e).

### 3.3 PPTT of hCFs Loaded with AuNRs

Adherent hCFs were treated with 50 μM, 100 μM, and 200 μM AuNRs for 72 h, detached using enzymatic digestion, suspended in PBS 2% FBS in a quartz cuvette, and then top-illuminated. To establish an appropriate photothermal treatment plan, 10 min of illumination time was selected to guarantee a uniform heating distribution across the volume of the sample (Figure 4a). The temperature variations of AuNR-loaded cell samples as a function of the irradiation time were measured (Figure 4b). Thermal profiles revealed a correlation between the increase in the concentration of AuNRs used to treat the cells and the temperature variation (Figure 4c), calculated as Δ*T* = *T*_max_ – *T*_min_, thus showing a trend in agreement with the temperature–time profiles previously measured. The effect of irradiation on cell viability was assessed using flow cytometry, which revealed a dose-dependent increase in the percentage of both necrotic and late apoptotic cells (Figure 4d) labeled as AnnV-/PI+ and AnnV+/PI+, respectively (Figure 4e–h). This outcome is consistent with previous studies, which have demonstrated that the heat generated during photothermal therapy typically causes membrane disruption and necrotic cell death.^1,3,6^

**Figure 4:**
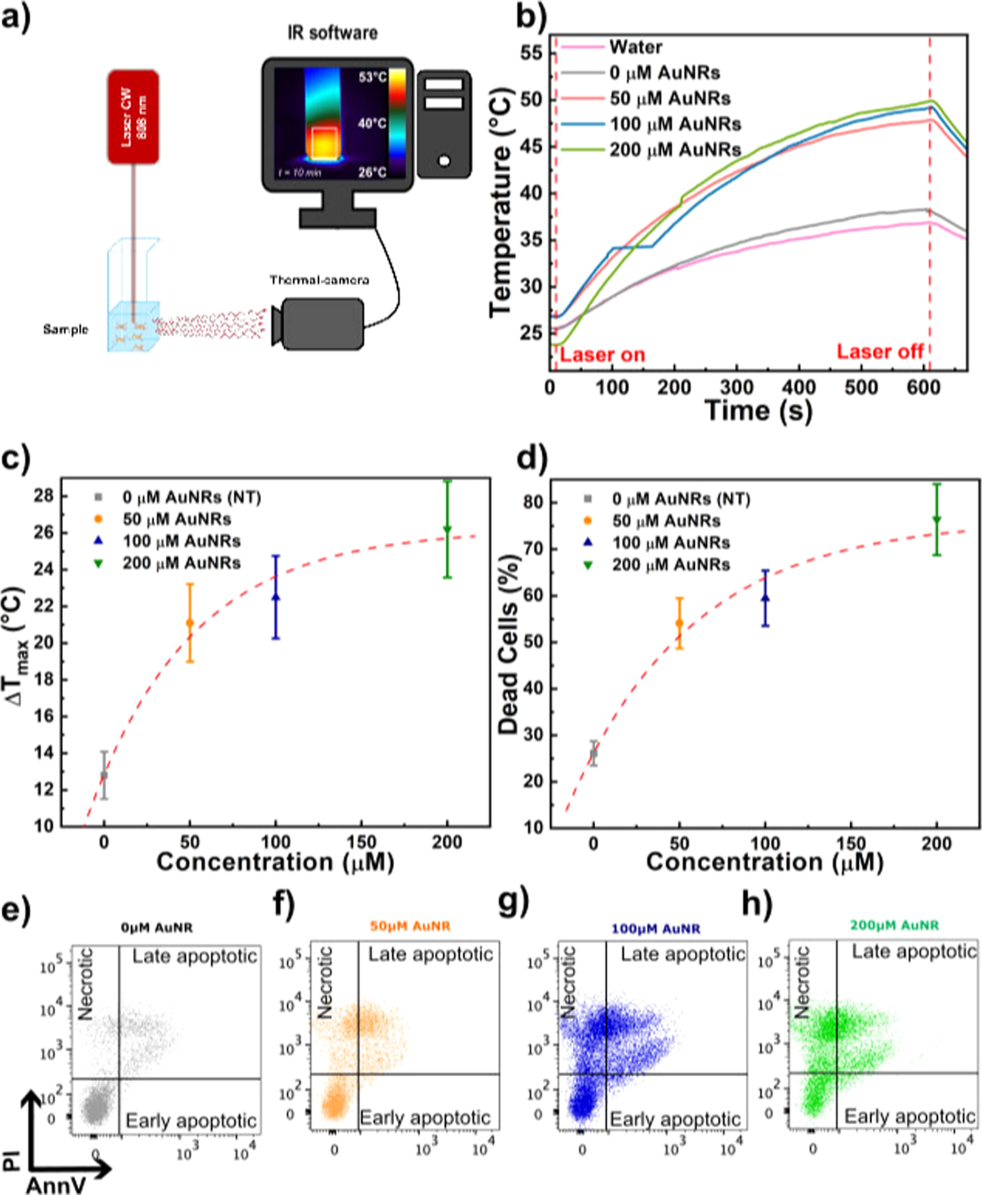
Optical irradiation and viability of AuNR-loaded hCFs. (a) Optical and thermal setup design for irradiation in suspension. In the inset is shown the representative thermal profile of a cell solution with 100 μM AuNRs after 10 min of irradiation. (b) Comparison of the temperature–time profiles of the different cell samples with concentration 0, 50, 100, 200 μM, and water, used as a control measurement. The temperature variations are Δ*T* (50 μM) = 21.5 °C, Δ*T* (100 μM) = 22.5 °C, Δ*T* (200 μM) = 26.2 °C, Δ*T* (0 μM) = 12.8 °C and Δ*T* (water) = 11.4 °C. (c) Correlation plot of the temperature reached by the samples following illumination as a function of AuNRs concentration. (d) Correlation plot of the percentage of dead cells (both necrotic and late apoptotic) following illumination with a CW diode laser operating at 808 nm for 10 min, as a function of AuNPs concentration. (e–h) Representative dot plots of flow cytometry analysis for AnnexinV and Propidium Iodide staining are shown.

### 3.4 Thermal Ablation of AuNR-Loaded hCFs via PPTT in Mixed Cocultures

The ability of PPTT-mediated ablation of the sole AuNR-loaded CFs, without damaging surrounding nonloaded cells, was evaluated using an in vitro coculture system. First, culture and illumination setup for adherent cell cultures was optimized for this set of experiments, and tested for the detection of temperature variations and cell death within the imaging system (Figure 5a). The hCFs were pretreated with 100 μM AuNRs for 72 h, plated to adhere on a culture slide and irradiated. Cell death was assessed using a live/dead fluorescent assay. Fluorescence imaging of nonloaded hCFs (CTR) with or without irradiation (“laser on” and “laser off”, respectively), as well as nonirradiated AuNR-loaded hCFs (AuNR-hCFs), show only live cells (calcein-positive) (Figure 5b–d). AuNR-hCFs irradiated for 10 min reveal the death of all the cells (EthDIII-positive), confirming the efficacy of photothermal ablation in this setup (Figure 5e).

**Figure 5:**
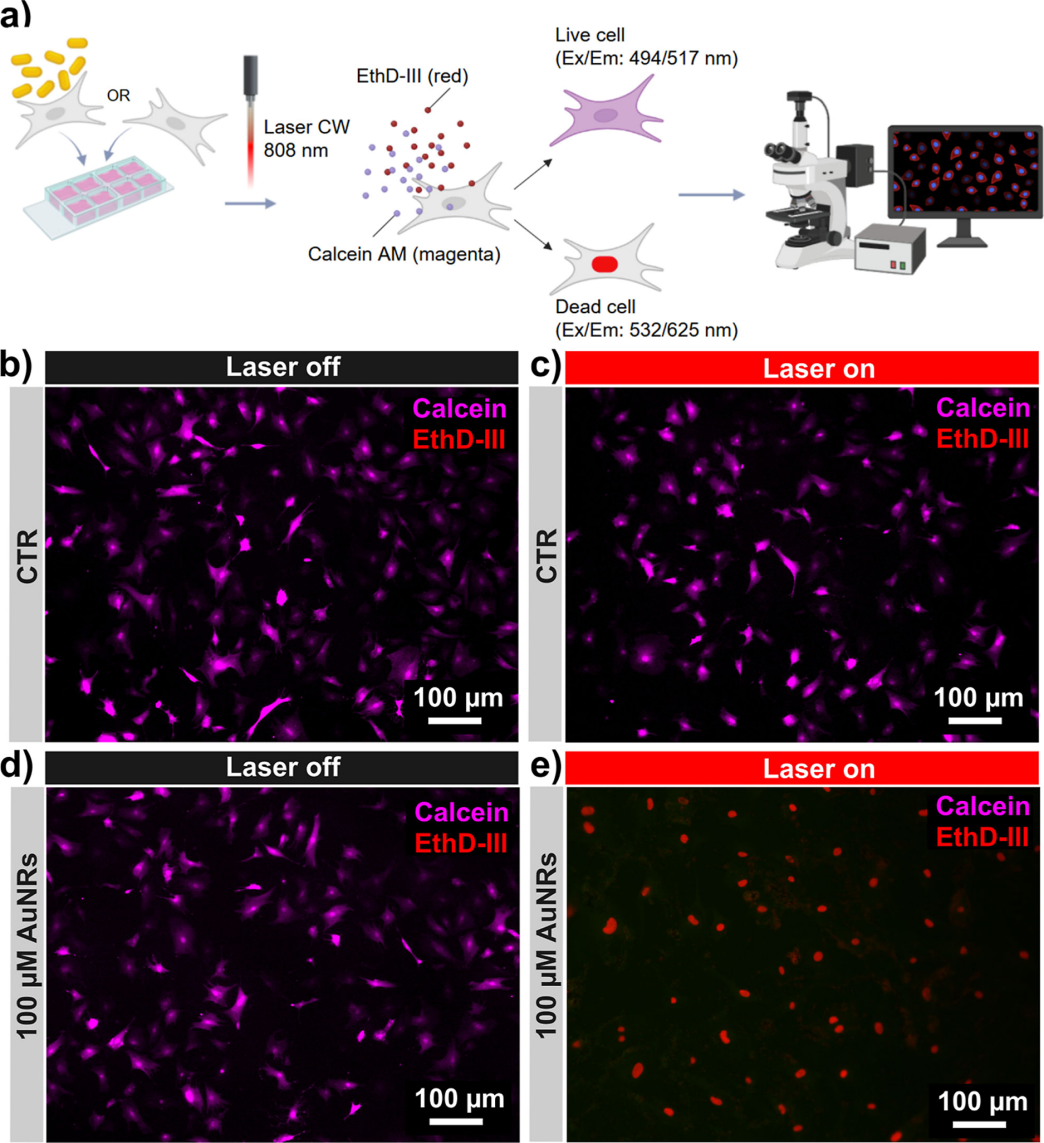
Optimization of the irradiation setup for adherent cell cultures. (a) Workflow depiction of the seeding, irradiation, and cell viability evaluation protocol. The image was created using Biorender.com. Representative fluorescence images of the samples, pretreated (d,e) or not (b,c) with 100 μM AuNRs for 72 h, and then either irradiated (c,e) or not (b,d) with a CW diode laser at 808 nm for 10 min. Viable hCFs are calcein-positive (purple fluorescence) while dead hCFs are ethidium-positive (red fluorescence). CTR = untreated control.

Next, the cellular system designed to test the efficacy of ablating only AuNR-loaded CFs was composed of a mixed adherent culture including hCFs pretreated for 72 h with 100 μM AuNRs (AuNR-CFs) and nontreated GFP-expressing hCFs (hCF^GFP^) (Figure 6a). The hCF^GFP^ lines were tested to ensure that GFP expression would not affect the absorption spectra of the cells, as well as the efficacy of AuNR treatment in inducing cell death after irradiation (Figure S1f–l). The culture was set up with a 50% ratio of AuNR-hCF/hCF^GFP^ (Figure 6a,b) and irradiated for 10 min. The temperature variation measured was Δ*T* = 4.2 °C (Figure 6c). Cell death was assessed by fluorescent live/dead assay, showing that only the AuNR-hCFs were dead (GFP negative-EthDIII-positive), while the viability of nonloaded hCF^GFP^ was preserved as indicated by the absence of GFP positive-EthDIII positive nuclei within hCF^GFP^ cells in the culture (Figure 6d,e).

**Figure 6:**
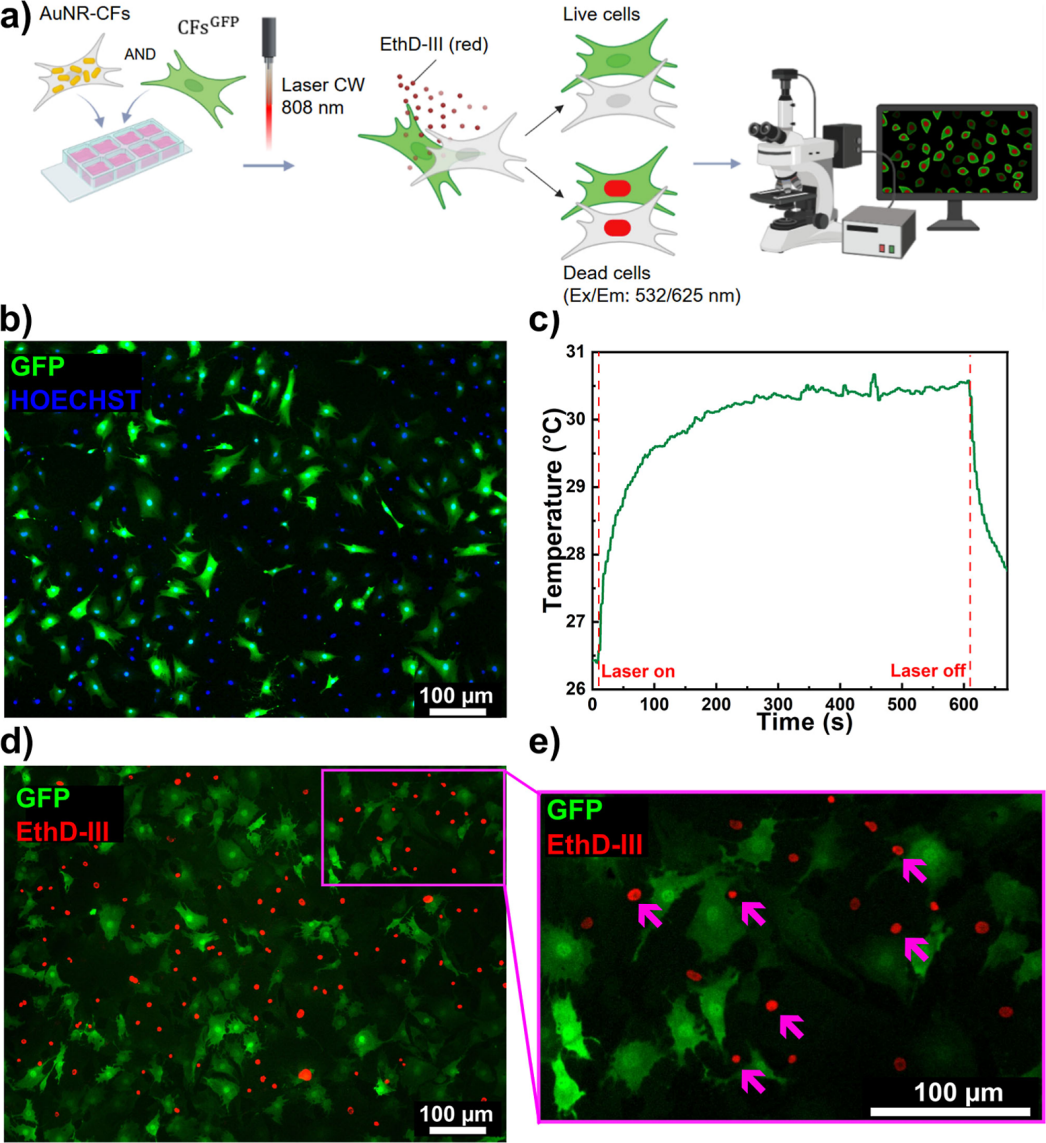
Irradiation experiment on the coculture with AuNR-loaded and nonloaded hCFs. (a) Experimental design of the mixed coculture establishment, irradiation, and cell viability assessment. The image was created using Biorender.com. (b) Representative fluorescence image of the mixed coculture of 50% hCFs pretreated with 100 μM AuNRs for 72 h (GFP-negative) and 50% nontreated hCFs^GFP^. (c) Temperature–time profile of the mixed coculture irradiated with a CW diode laser (Coherent Powerline) operating at 808 nm for 10 min. (d) Representative fluorescence image of hCFs on culture slides after irradiation of the mixed coculture, and (e) higher magnification panel. Cell death staining (red fluorescence, ethidium-positive) can be detected only in hCFs not expressing GFP, identifying AuNR-treated cells, while hCFs^GFP^ never show red nuclei. Blue fluorescence: HOECHST, nuclei. Red fluorescence: EthD-III, dead cells. Green fluorescence: GFP.

To evaluate the distance at which cellular ablation due to intracellular heating induced by irradiated AuNRs could be extended to surrounding cells, the minimum distance between the nuclei of live and dead cells was measured after illumination of the AuNR-hCF/hCF^GFP^ coculture. The closest living cell was evaluated for each dead cell, and the distance between the nuclei was calculated. The distribution of the minimum distances between the nuclei of living cells and the nuclei of dead cells (Figure S1m) showed that the minimum distance is *d*_min_ = 10.72 μm, with a mean measured distance of *d*_mean_ = 35.08 ± 14.30 μm, where the error was estimated using the calculated standard deviation.

The selective ablation of the sole AuNR-loaded cells was also assessed in a physiologically relevant model consisting of CFs and HL-1 cardiomyocytes. The AuNR-CFs were mixed in suspension with HL-1^GFP^ cardiomyocytes. The cell suspension was top-irradiated in a quartz cuvette for 10 min after the sample had reached the maximum temperature of 46 °C, considered as a moderate temperature increase necessary for the achievement of cell death.^1,43^ The temperature variation was measured, and cell death of both AuNR-CFs and HL-1^GFP^ was assessed using flow cytometry analysis of AnnV and PI staining (Figure 7a). The temperature variations reached a maximum of Δ*T* = 17.9 (Figure 7b). The percentage of live cells (AnnV-/PI-) was significantly reduced in AuNR-CFs after irradiation, while it was preserved in HL-1^GFP^ (Figure 7c). In detail, the proportion of necrotic (AnnV-/PI+) and late apoptotic (AnnV+/PI+) AuNR-CFs was significantly higher compared to the HL-1^GFP^ cells (Figure 7d,e).

**Figure 7:**
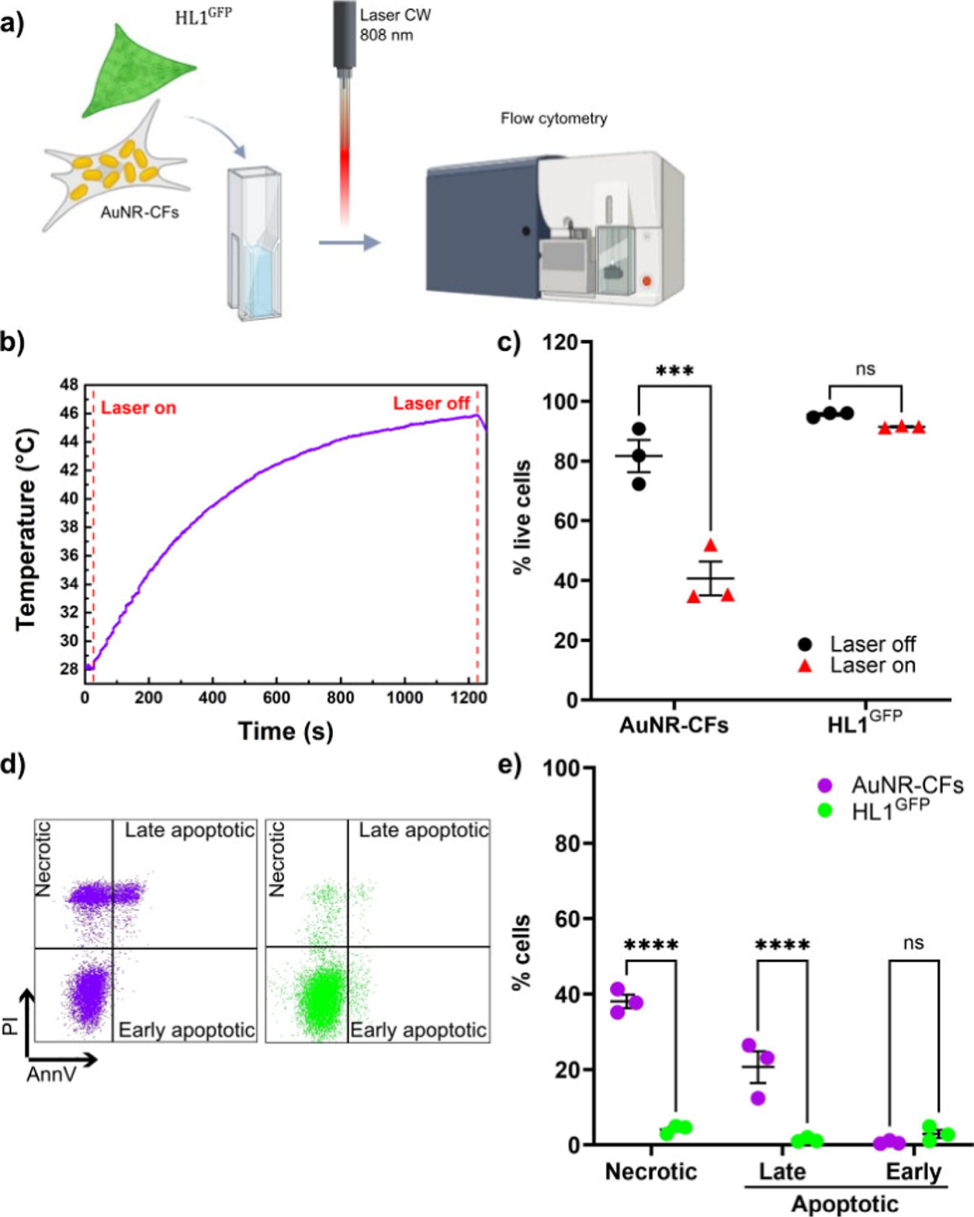
Irradiation experiment on the coculture with AuNR-loaded hCFs and nonloaded HL-1 cardiomyocytes. (a) Experimental design of the multicellular cell suspension, with irradiation, and cell viability assessment. The mixed cell suspension was composed of CFs treated with AuNRs for 72 h (AuNR-CFs) and nontreated HL-1^GFP^ cardiomyocytes. The image was created using Biorender.com. (b) Representative temperature–time profile of the mixed cell suspension irradiated with a CW diode laser (Coherent Powerline) operating at 808 nm. (c) Dot plot showing the percentage of live cells (AnnV-/PI-) within AuNR-CFs and HL-1^GFP^, identified through flow cytometry analysis of the multicellular cell suspension after irradiation. The percentage of live cells (AnnV-/PI-) was significantly reduced in AuNR-CFs after irradiation (40.67 ± 5.67%). (d) Representative plots of flow cytometry gating strategy for the evaluation of AuNR-CFs and HL-1^GFP^ viability. (e) Dot plot showing the percentage of necrotic (AnnV-/PI+), late apoptotic (AnnV+/PI+), and early apoptotic (AnnV+/PI-) AuNR-CFs and HL-1^GFP^ after irradiation of the mixed cell suspension. The proportion of necrotic (AnnV-/PI+) and late apoptotic (AnnV+/PI+) AuNR-CFs is significantly higher (respectively 38.07 ± 1.77% and 20.67 ± 4.25%) compared to the HL-1^GFP^ cells (4.19 ± 0.64% and 1.33 ± 0.37%). *n* = 3; NS: nonsignificant. ***: *p* < 0.001. ****: *p* < 0.00001.

### 3.5 Discussion

PPTT is a promising noninvasive and drug-free approach for the specific ablation of detrimental or pathological cells in tissues for therapeutic purposes, achieved by producing intracellular heat through NPs irradiation with NIR light. Due to its inertness, colloidal stability, and biocompatibility, gold is often used to fabricate plasmonic NPs for biological applications. So far, AuNP-mediated PPTT has found potential applications primarily for the treatment of cancer and microbial infections.

Cardiac fibrosis is a pathological condition, commonly seen across several cardiovascular diseases, characterized by excessive collagen production and deposition in the myocardial tissue interstitium in response to cellular damage and inflammatory stimuli. It results in the formation of nonfunctional scar tissue and stiffening of the myocardial muscle, progressively causing cardiac dysfunction. The central effectors in this process are hCFs, which become activated after injury and produce ECM proteins. Although scar formation is a fundamental repair mechanism which avoids organ rupture, multiple studies have recently shown that a partial blockage of this response can produce beneficial effects on the extension of the damaged area, and preserve cardiac function. As evidenced by nanoparticle characterization, AuNRs exhibit dimensions and morphological features suitable for uptake by CFs and have been demonstrated to be efficient thermo-optical transducers under NIR irradiation (Figure 2).

AuNRs loading of CFs demonstrated high biocompatibility since cell viability and subcellular organization were unaffected by treatment at increasing dosage of AuNRs (Figure 3b,c). The subcellular distribution of AuNRs in hCFs showed the uptake of AuNRs in endocytic vacuoles. This uptake was accompanied by unique absorbance spectra, showing a redshift of the longitudinal peak, consistent with AuNRs internalization (Figure 3e,f). Ultrastructural analysis using TEM was utilized as the preferred technique to examine the interactions of nanoparticles with the biological environment (Figure 3e,h). The experimental data indicated a strong correlation between the optical and morphological characterization of the internalized AuNRs in hCFs. The observed redshift and broadening of the longitudinal plasmon band serve as indirect evidence of AuNR clusters forming within the cellular vacuoles, which was confirmed through TEM analysis. Our results using TEM show that AuNRs uptake ends in defined subcellular structures, consistent with the literature reporting that nanocarriers can interact with the plasma membrane, where they are internalized through membrane invaginations, entering the cell enclosed in endosomes.^44–46^ In our study, AuNR remnants persist within vacuolar structures in a clustered form, with precise compartmentalization observed. The cytoplasm of these cells was predominantly occupied by large vacuoles (V), reducing the presence of other organelles, such as mitochondria (m) and the Golgi apparatus. However, the cytoplasm contained a well-developed rough endoplasmic reticulum (rER) with regular cisternae, in line with the normal fibroblast-like morphology and their typical physiological activity. Overall, our data suggest that no morphological markers of cellular stress, apoptosis, or autophagy are observed following treatment with AuNRs and their subsequent internalization.

This study reports that irradiation of primary hCFs loaded with AuNRs can yield selective ablation of AuNR-containing cells without affecting bystander cells at a scale relevant to tissue physiopathology. As evidenced by nanoparticle characterization, AuNRs exhibit dimensions and morphological features suitable for uptake by hCFs and have been demonstrated to be efficient thermo-optical transducers under NIR irradiation. NIR light has a penetration depth of 3 mm; therefore, it can be envisaged as a potential in situ application for PPTT on fibrotic tissues. The longitudinal plasmon band of AuNRs selected for this work perfectly matched the NIR laser wavelength, resulting in effective and efficient thermo-optical transduction. This is a crucial point since PPTT can grant high specificity through multiple levels of control: first, at the biological level, by the design of cell-type-specific AuNPs for selective uptake; second, at the spatial level, by the spatially controlled irradiation process on the target tissue area; and third, at the desired timing of therapeutic intervention.

PPTT of AuNR-treated hCFs in suspension causes a temperature increase that leads to cell death, with an extent proportional to the AuNR concentration at a fixed laser power density, suggesting that the simple parameter of AuNR concentration can be modulated to obtain different yields and depletion efficacy under a constant irradiation source (Figure 4). When mixed with nontreated cells, sample irradiation mediates the death of only the AuNR-loaded hCFs, without any bystander effect on the viability of surrounding cells, including other CFs and cardiomyocytes, within an average distance of 35 μm (Figure 6). Selective ablation was evaluated in a physiologically relevant environment using CF/HL-1 cardiomyocyte coculture, where AuNR-loaded CFs were irradiated at a moderate hyperthermic temperature (46 °C), reaching a maximum Δ*T* of 17.9 °C. Flow cytometry revealed a significant loss of viability and increased necrotic and late apoptotic fractions in AuNR-CFs, while HL-1^GFP^ cardiomyocytes remained largely unaffected (Figure 7).

This result is significant for the potential safety of the approach on bystander nontargeted cardiac cells, such as cardiomyocytes, or other stromal nontargeted cell populations. Future studies on more complex 3D models will be needed to corroborate this conclusion.

## 4 Conclusions

The present work explores an approach for treating cardiac fibrosis based on selective cellular ablation through PPTT mediated by AuNRs. This approach was extensively investigated for cancer treatment and was explored here for the first time in a cell type highly relevant to cardiac fibrosis. In particular, the interaction of AuNRs with primary hCFs was demonstrated, taking into account AuNRs internalization, cell viability, and the optical properties of the AuNR-loaded cellular system. Ultrastructural analysis performed by TEM demonstrated internalization of AuNRs, while a detailed photothermal investigation demonstrated that AuNRs generate a photothermal heating able to induce hCF cell death. Remarkably, the selectivity of the PPTT approach toward AuNR-loaded CFs was demonstrated in a coculture system with cells not treated with AuNRs, traceable via GFP constitutive expression. Our results show that there is no detrimental bystander effect from heating irradiated AuNR-loaded cells, in both suspension and 2D culture settings. Overall, in this system, the heat generated by NIR illumination is sufficient to selectively kill all cells that have internalized AuNRs without damaging the unloaded surrounding cells, at a scale potentially significant for in situ applications. Based on the results obtained, the proposed approach opens new avenues for developing a minimally invasive technique capable of targeting specific cardiac interstitial cells with predictably low side effects. As a future development of this approach, AuNRs could be functionalized with specific antibodies or peptides that recognize and bind surface markers selectively expressed on activated CFs during the fibrotic process, enabling targeted cellular uptake and selective delivery to pro-fibrotic or pro-inflammatory CF subtypes. In vivo studies in mouse models of cardiac fibrosis will be essential to evaluate different routes of administration and to optimize cardiac tropism. A dedicated thermo-optical setup will be developed, and irradiation parameters, such as laser wavelength, intensity, and exposure time, will be adjusted to achieve precise photothermal ablation of activated fibroblasts and consequent reduction of fibrotic remodeling. Once the therapeutic efficacy and safety of this strategy are established in vivo, further refinements could pave the way for its translational application in humans, accompanied by the development of innovative, minimally invasive, biotransparent laser sources for NIR irradiation, which will provide a novel, selective photothermal therapy for the treatment of cardiac fibrosis. This represents a significant advancement in nanomedicine, expanding its therapeutic benefits for treating fibrosis associated with several cardiovascular diseases.

## Supplementary Material



## Notes

Supplementary experimental results, additional materials and methods, and photographs of the experimental setup, including supplementary Figures S1 and S2 with associated captions (PDF)
