# Supplementary material for: Killing of Gold Nanorods-Loaded Human Cardiac Fibroblasts Mediated by Photo-Thermal Activation
Source: ACS Omega. 2026 Feb 4;11(6):10589–602. doi: 10.1021/acsomega.5c12235 (PMC12917637; doi:10.1021/acsomega.5c12235)
Supplement: Supplementary file 1 [file ao5c12235_si_001.pdf]

SUPPORTING INFORMATION

Killing of Gold Nanorods-Loaded Human Cardiac Fibroblasts Mediated by Photo-Thermal Activation

Erica Floris[a], *Flaminia Pompeo*[b], *Vittorio Picchio*[c], *Selenia Miglietta*[d], *Vincenzo De Mei*[a], *Claudia Cozzolino*[a], *Francesca Icolaro*[a], *Vincenzo Petrozza*[a], *Giacomo Frati*[a,c], *Francesca Petronella*[b # *], *Isotta Chimenti*[a,e # *], *Francesca Pagano*[f # *], *Luciano De Sio*[a # *].

a Department of Medical Surgical Sciences and Biotechnologies, Sapienza University of Rome, Corso della Repubblica 79, 04100 Latina, Italy

b Institute of Crystallography CNR-IC, Institute of Crystallography CNR-IC, Rome Division, Area della Ricerca Roma 1, Strada Provinciale 35d, 00010 Montelibretti (RM), Italy

c Department of Angio-Cardio-Neurology, IRCCS Neuromed, Via Atinense 18, 86077 Pozzilli (IS), Italy

d Department of Anatomy, Histology, Forensic Medicine and Orthopaedics, Sapienza University of Rome, Via A. Borelli 50, 00161 Rome, Italy

e Maria Cecilia Hospital, GVM Care & Research, Via Corriera 1, 48033 Cotignola (RA), Italy

f National Research Council of Italy, Institute of Biochemistry and Cell Biology, Via E. Ramarini 32, 00015 Monterotondo Scalo (RM), Italy

Corresponding Authors:

Francesca Petronella: francesca.petronella@cnr.it

Isotta Chimenti: isotta.chimenti@uniroma1.it

Francesca Pagano: francesca.pagano@cnr.it

Luciano De Sio: luciano.desio@uniroma1.it

# F.Pe., I.C., F.Pa., L.D.S. contributed equally to this work

SUPPORTING INFORMATION:

Supplementary experimental results, additional materials and methods, and photographs of the experimental setup, including supplementary Figures S1 and S2 with associated captions

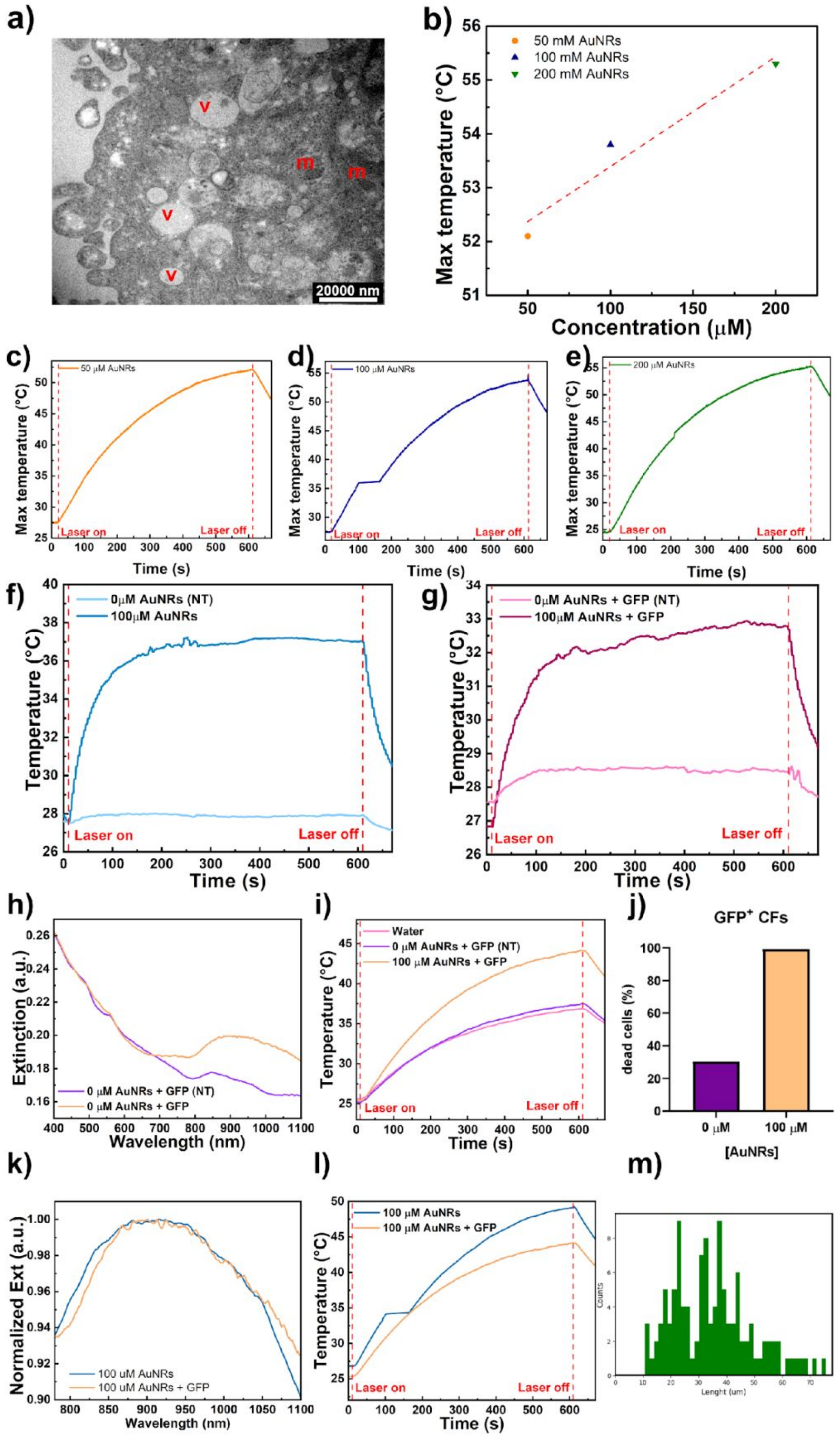

Figure S1: a) Representative ultrastructural TEM images of the hCFs. b) Correlation plot of the maximum temperature reached by the samples following illumination as a function of AuNRs concentration. The maximum temperature are: Tmax(50 μM)= 52.1 ˚C, T(100 μM)= 53.8 ˚C, T(200 μM)= 55.3 ˚C. Maximum temperature-time profiles of the different cell samples with concentration

50 μM (c), 100 μM (d), 200 μM (e). f) Comparison between the temperature-time profiles of cell samples on a slide, with concentration 0 and 100 μM. The temperature variations are ΔT(100 μM)= 9.4 ˚C, ΔT(0 μM)= 0.4 ˚C. g) Comparison between the temperature-time profiles of GFP-cell samples on a slide, with concentration 0 and 100 μM. The temperature variations are ΔT(100 μM)= 6.0 ˚C, ΔT(0 μM)= 1.1 ˚C. h) Comparison between the absorption spectra of the samples. It is noted that in the sample without AuNRs there is no absorption peak, while in the sample with AuNRs there is a peak in λmax(100 μM GFP)= 913 nm. i) Comparison between the temperature-time profiles of GFP-cell samples with concentration 0 and 100 μM and water, used as a control measurement. The temperature variations are ΔT(100 μM)= 18.8 ˚C, ΔT(0 μM)= 12.0 ˚C and ΔT(water)= 11.4 ˚C. j) Viability of GFP+ hCFs treated with 0 μM and 100 μM AuNRs for 72 hours, and irradiated with a CW diode laser operating at 808 nm for 10 minutes. Cells were stained for AnnexinV and Propidium Iodide, and analysed through flow cytometry. k) Comparison between the normalized absorption spectra of the cell samples with and without GFP. l) Comparison between the temperature-time profiles of cell samples with and without GFP. The temperature variations are ΔT(100 μM)= 22.5 ˚C and ΔT(100 μM GFP)= 18.8 ˚C. m) Distribution of minimum distances between live and dead cells in the mixed co-culture setting.

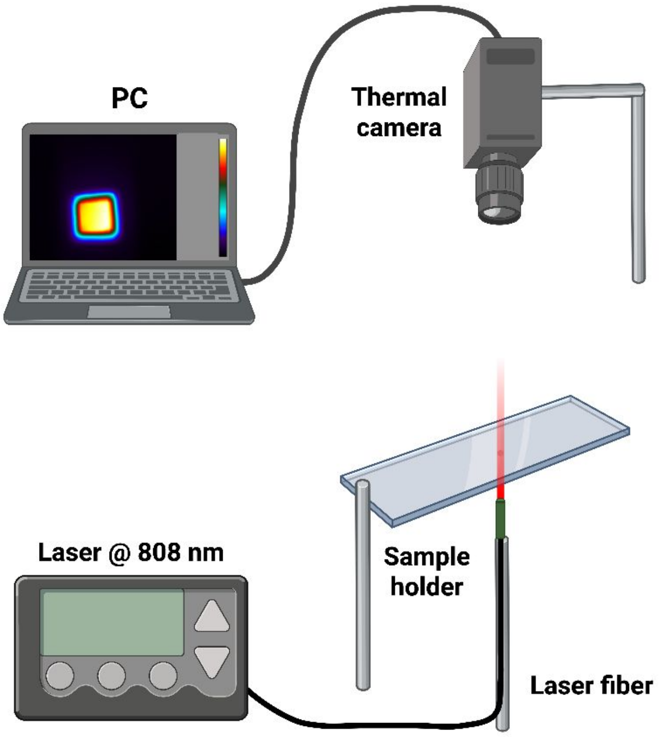

Figure S2: Schematic of the photo-thermal experimental setup employed to study mixed co-culture samples, showing bottom-side laser irradiation and top-side thermal imaging. Created in BioRender. De Mei, V. (2026) https://BioRender.com/u3jeyjj
